# Neonatal Screening and Genotype-Phenotype Correlation of 21-Hydroxylase Deficiency in the Chinese Population

**DOI:** 10.3389/fgene.2020.623125

**Published:** 2021-01-22

**Authors:** Xin Wang, Yanyun Wang, Dingyuan Ma, Zhilei Zhang, Yahong Li, Peiying Yang, Yun Sun, Tao Jiang

**Affiliations:** Genetic Medicine Center, Women's Hospital of Nanjing Medical University, Nanjing Maternity and Child Health Care Hospital, Nanjing, China

**Keywords:** congenital adrenal hyperplasia, 21-hydroxylase deficiency, neonatal screening, genotype-phenotype, CYP21A2

## Abstract

**Background:** Congenital adrenal hyperplasia (CAH) is a group of autosomal recessive disorders encompassing enzyme deficiencies in the adrenal steroidogenesis pathway that leads to impaired cortisol biosynthesis. 21-hydroxylase deficiency (21-OHD) is the most common type of CAH. Severe cases of 21-OHD may result in death during the neonatal or infancy periods or sterility in later life. The early detection and timely treatment of 21-OHD are essential. This study aimed to summarize the clinical and genotype characteristics of 21-OHD patients detected by neonatal screening in Nanjing, Jiangsu province of China from 2000 to 2019.

**Methods:** Through a retrospective analysis of medical records, the clinical presentations, laboratory data, and molecular characteristics of 21-OHD patients detected by neonatal screening were evaluated.

**Results:** Of the 1,211,322 newborns who were screened, 62 cases were diagnosed with 21-OHD with an incidence of 1:19858. 58 patients were identified with the classical salt-wasting type (SW) 21-OHD and four patients were identified with simple virilizing type (SV) 21-OHD. Amongst these patients, 19 cases patients accepted genetic analysis, and another 40 cases were received from other cities in Eastern China. Eighteen different variants were found in the *CYP21A2* gene. The most frequent variants was c.293-13A/C>G (36.29%). The most severe clinical manifestations were caused by large deletions or conversions of *CYP21A2*.

**Conclusions:** This study suggested that neonatal screening effectively leads to the early diagnosis of 21-OHD and reduces fatal adrenal crisis. Our data provide additional information on the occurrence and genotype-phenotype correlation of 21-OHD in the Chinese population which can be used to better inform treatment and improve prognosis.

## Introduction

Congenital adrenal hyperplasia (CAH) (OMIM# 201910) comprises a family of autosomal recessive disorders that are characterized by a group of enzymatic defects in cortisol biosynthesis due to defects in the steroid 21-hydroxylase gene (*CYP21A2*, OMIM^*^ 613815) (Nimkarn et al., 1993; Baumgartner-Parzer et al., 2020). Impaired cortisol production removes negative feedback control on the hypothalamus and pituitary glands that acts to increase the secretion of corticotropin-releasing hormone (CRH) and adrenocorticotropic hormone (ACTH) resulting in hyperplasia of the adrenal cortex (Merke and Bornstein, 2005). Steroid 21-hydroxylase deficiency (21-OHD OMIM: 201910) accounts for over 95% of CAH cases (Speiser et al., 2010, 2018; El-Maouche et al., 2017). The impairment of mineral corticoid synthesis causes adrenal crises and electrolyte disorders in infants (Parsa and New, 2017).

21-OHD can be classified as salt-wasting (SW), simple virilizing (SV), and non-classical (NC) types in neonates. SW type is the most severe form of 21-OHD that is responsible for ~75% of 21-OHD cases. SV type disease accounts for around 25% of cases, whilst NC type is rare in the clinic and accounts for <1% of cases (Parsa and New, 2017). The major clinical manifestations of 21-OHD include hyperpigmentation, ambiguous reproductive organs in female neonates, precocious puberty in male neonates, premature pubarche in children, and short stature in adulthood (Witchel, 2017). In addition to the above symptoms, patients with SW type disease who do not receive treatment may experience electrolyte disorders, hypovolemia and shock due to aldosterone deficiency. Severe cases may even result in death during the neonatal or infancy periods (White, 2009; Gidlöf et al., 2014). As patients show no initial symptoms, NC type disease can be manifested as hyperandrogenism that results in sterility in later life (Parsa and New, 2017).

Clinical evidence has shown ambiguous genitalia mainly in female patients at birth or SW symptoms such as feeding intolerance, vomiting, diarrhea, and skin pigmentation during the neonatal period can lead to the suspicion of 21-OHD. In male patients, particularly those with atypical SW symptoms, 21-OHD can be easily misdiagnosed and so elevated 17-hydroxyprogesterone (17-OHP) levels remain the best biomarker for early diagnosis (Jailer et al., 1955; Miller, 2019). Early diagnosis and long-term standardized treatments can reduce mortality and greatly improve the prognosis of 21-OHD patients (Grosse and Van Vliet, 2007).

This study aimed to characterize the clinical phenotypes and genotype data of 21-OHD patients. We analyzed and summarized the clinical data from the neonatal screening of 17-OHP in China over 20 years to gain insight into the occurrence and genetics of 21-OHD to better inform treatment and improve prognosis.

## Materials and Methods

### Patients

We screened 1211322 neonates born in Nanjing, Jiangsu province of China from January 2000 to December 2019, among the screen-positive newborns, 62 were diagnosed as 21-OHD. They all came from unrelated families.

This study was approved by the Ethics Committee of Nanjing Maternity and Child Health Care Hospital affiliated with Nanjing Medical University. All the parents of participating neonates provided written informed consent.

### Neonatal Screening of 17-OHP

When the neonates were 48–72 h after birth with full lactation, 200 μL of heel blood was collected to create a dry blood filter paper. The concentrations of 17-OHP were measured using time-resolved immunofluorescence with a cut-off level of 30 nmol/L from 2000 to 2018 (Wallac 1420: Januar 2000 to December 2013; AutoDEFIA Wallac1235: January 2014 to Octobe 2018, Turku, Finland). From October 2018 to the present day, the cut-off value of 12 nmol/L was established by our laboratory obtained through the analysis of a percentile method combined with ROC curve (Wallac 2021-0010: October 2018 to now). The assay kits were purchased from Perkin Elmer (B015:2000–2013; B016: 2014–2018; B024: 2018 to now, Turku, Finland).

### Clinical Diagnosis

Cases with 17-OHP levels greater than the cut-off values (30 nmol/L from January 2000 to October 2018; 12 nmol/L from October 2018 to the present day) twice were suspicious 21-OHD (Cases with low birth weight or premature delivery. If the recall review was still higher than the normal range, sample were retested for the third time when the correct gestational ages were close to 40 weeks or the weights were more than 2500 g). For suspicious cases, blood biochemical criteria (electrolysis quality, ACTH, cortisol, testosterone) were determined and genetic analysis performed. Definitive diagnosis was made base on abnormal biochemical criteria, gene analysis, and clinical manifestation such as hyperpigmentation, vomiting, dehydration, hypotension, hyponatremia, hyperkalemia, a lack of weight gain, shock, and ambiguous genitalia according to the consensus statement on diagnosis and treatment of congenital adrenal hyperplasia due to 21-OHD (Subspecialty Group of Endocrinologic, 2016).

### Treatment and Follow-Up

Once the clinical diagnosis is clear, hydrocortisone acetate 15–20 mg/m^2^/day should be given immediately, and 9α flurocortisone 0.05–0.15mg/day should be added to SW. Hydrocortisone 8–10mg/ kg should be given intravenously to patients with cortical functional crisis (It is mainly neonatal period) 2–3 times a day and they should be taken orally after the crisis is relieved. During the follow-up, Blood electrolyte, 17-OHP, plasma electrolysis quality and adrenocorticotropic hormone were reexamined every 3 months, bone age was reexamined every 12 months to adjust the treatment dose. The dosage of hydrocortisone acetate increased by 2–3 times when the cases had a fever, diarrhea and other infections. Female patients with hermaphroditism usually complete clitoral orthopedics before 2 years old. Some cases especially with SW may use growth hormone or Tamiflu before puberty.

### Locus-Specific PCR

QIAamp DNA blood kits (Qiagen, Venlo, The Netherlands) were used to extract genomic DNA from 3 to 5ml of anticoagulated peripheral blood samples. *CYP21A2* rearrangement products were confirmed by locus-specific PCR. Four primers were designed which specific located upstream and downstream of either *CYP21A2* (ME0008 and ME0066) or CYP21A1P (ME0059 and ME0067) (Lee et al., 1996; Keen-Kim et al., 2005), and the specific PCR products obtained were shown in Supplementary Table 2. The 50 μL reaction mixture contained 100 ng DNA template, 1 × GC Buffer I, 0.4 mM dNTP, 0.4 μM of each primer, and 2.5 U LA Taq DNA polymerase (TaKaRa, Dalian, China). The PCR amplification conditions were: 94°C for 1 min, then 35 cycles at 94°C for 30 s, 60°C for 30 s, 72°C for 3 min, and a final extension at 72°C for 10 min. To ensure the specificity of each reaction, we performed restriction endonuclease analysis of the four PCR amplicons. After PCR, each of the four products was digested with EcoRI (TaKaRa, Dalian, China) for 3 h at 37°C and the digested products were analyzed by electrophoresis on a 1.0% agarose gel. The *CYP21A2* gene contains one EcoRI site in intron 4, while the CYP21A1P pseudogene has two EcoRI sites in intron 2 and exon 4. The EcoRI digestion pattern of amplicons 3 and 4 depended on the location of the recombination breakpoint relative to the EcoRI site in intron 2.

### MLPA Analysis

Large gene deletions and conversions in the *CYP21A2* were identified by MLPA analysis using the SALSA MLPA kit (P050-B2 CAH, Amsterdam, Netherlands). This CAH probe mix contains 33 probes, including five probes for *CYP21A2* (exons 1, 3, 4, 6, and 8), three probes for CYP21A1P (exon 1, intron 2, and exon 10), 3 TNXB probes, 1 C4A probe, 1 C4B probe, 1 CREBL1 probe, 2 probes for chromosome 6p21.3, 1 UTY probe, and 16 reference probes. Hybridization, ligation, and amplification were performed according to the manufacturer's protocol. Amplification products were detected using an ABI 3130 Genetic Analyzer (Applied Biosystems, CA, USA) with LIZ500 (Applied Biosystems) as an internal size standard. The raw data were analyzed by using Coffalyser MLPA data analysis software (MRC Holland) (Ma et al., 2014). The *CYP21A2* mutations were named following Human Genome Variation Society nomenclature guideline (http://www.hgvs.org/mutnomen) by using RefSeq sequence (accession number: NM_000500.7).

### Statistical Analyses

Data are expressed as the median and range or the median ± standard deviation. An unpaired, two-way ANOVA test was used for between-group comparisons. Differences were considered significant when *P* < 0.05 (^*^P < 0.05; ^**^P < 0.01; ^***^P < 0.001).

## Results

### Neonatal Screening of 21-OHD During 2000–2019

**Figure 1:**
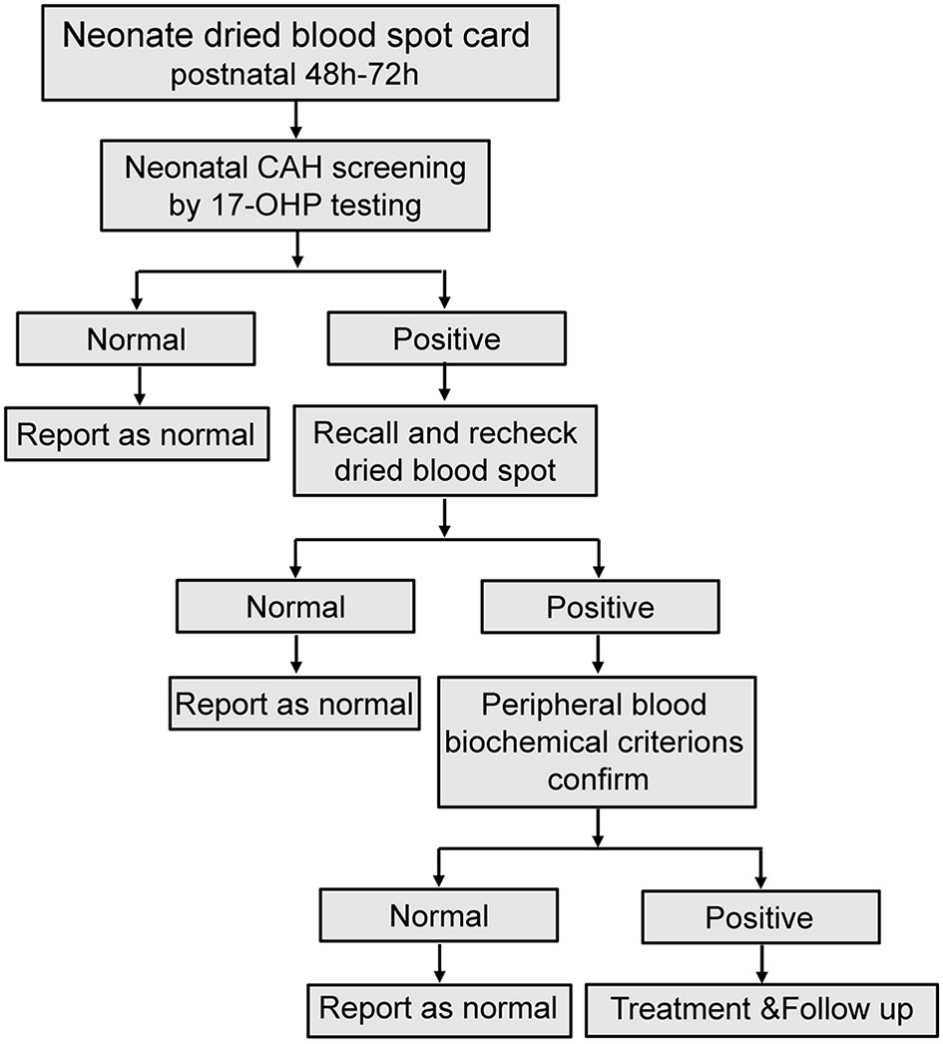
Flowchart for neonatal CAH screening. Recall and recheck dried blood spot recheck, re-analysis with another new dried blood spot from the recalled patient; Peripheral blood biochemical criterions confirm, analysis peripheral blood sample of biochemical criterions from recalled patient.

**Figure 2:**
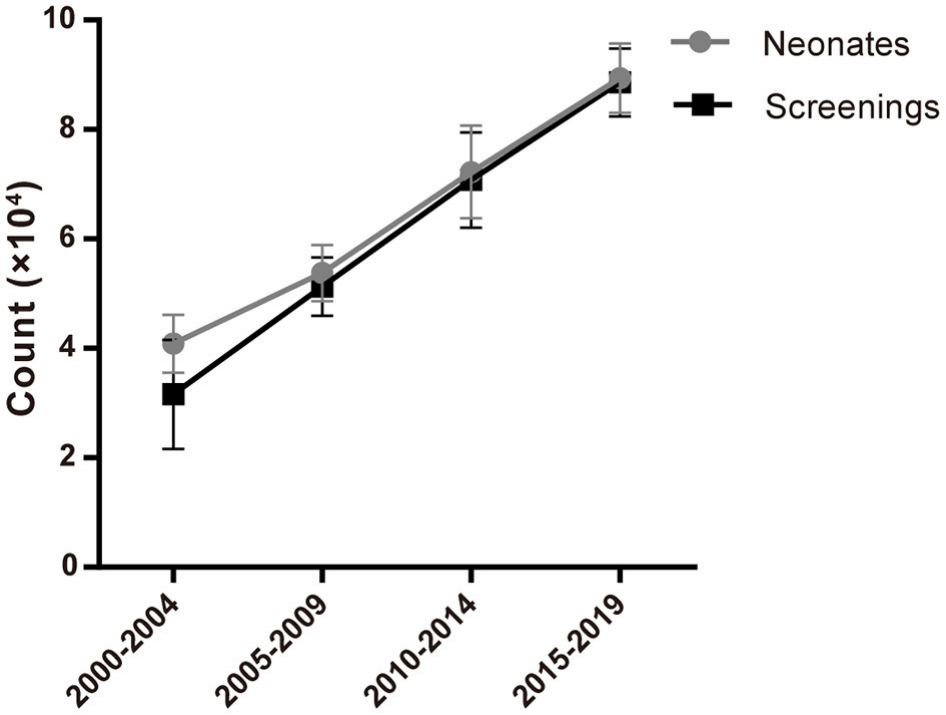
Number of neonates and screenings from 2000 to 2019.

1,211,322 neonates were screened for 21-OHD who were born in Nanjing, Jiangsu province of China from January 2000 to December 2019 (Figures 1, 2). Sixty-two cases of screen-positive newborns were diagnosed as 21-OHD including 58 SW cases (93.55%) (40 males, 18 females) and 4 SV cases (6.45%) (All males). NC cases were not found in this study. The incidence was one in 19,858 (Table 1) and the 17-OHP concentrations and other related indices are summarized in Tables 2, 3. The initial treatment ages of the neonates were 15 ± 6 days, and clinical follow-up after treatment showed that 17-OHP and other biochemical indicators were in the normal range.

**Table 1:** Statistical summary of 21-OHD incidence detected by neonatal screening.

**Time frame**	**Neonate**	**Screening**	**Positive**	**Incidence**
2000–2004	204275	157973 (77.33%)	4	1:39493 (0.00265%)
2005–2009	268834	256564 (95.44%)	15	1:17104 (0.00598%)
2010–2014	361188	353770 (97.95%)	19	1:18619 (0.00540%)
2015–2019	446762	443015 (99.16%)	24	1:18459 (0.00531%)
**2000**–**2019**	**1281059**	**1211322 (94.56%)**	**62**	**1:19537 (0.00512%)**

*Bold value indicates total analysis of data from 2000 to 2019*.

**Table 2:** Summary of 17-OHP concentrations from initial screening.

**Type**	**Cases (Males/Females)**	**Concentration nmol/L**	**Normal range nmol/L**
Salt wasting type	54 (36/18)	386.26 (45.70–1000)	0~30
	4 (4/0)	198.62 (102.47–350.60)	0~12
Simple virilizing type	4 (4/0)	52.7 (35.2–83.8)	0~30

*Concentration data are presented as the median (range)*.

**Table 3:** Laboratory findings for the 17-OHD patients at diagnosis.

**Hormone**	**Units**	**SW**	**SV**	**Normal range**
ACTH	pg/ml	185.46 ± 134.80	36.53 ± 23.28	0~46
Cortisol	ug/dl	4.76 ± 3.58	3.27 ± 2.13	4.26~24.86(am.)2.9~17.3(pm.)
Testosterone	ng/ml	9.12 ± 3.00	7.34 ± 2.42	1.42~9.23
K	mmol/L	5.98 ± 0.44	-	3.5~5.5
Na	mmol/L	126.03 ± 1.41	-	135~145
Cl	mmol/L	101.46 ± 8.31	-	96~108

*Data are presented as the median ± standard deviation*.

### *CYP21A2* Mutation Spectrum Analysis

Since 2014, genetic analysis has been widely used in neonatal screening, particularly for the diagnosis of 21-OHD patients who are suspected as CAH cases based on the presentation of clinical symptoms and biochemical analysis. Nineteen families with 21-OHD patients were born in Nanjing and 40 cases who had newborn screening in other cities in Eastern China and had then come to our hospital for further diagnosis and treatment. These patients accepted genetic analysis and came from unrelated families which none of the parents were consanguineous.

**Figure 3:**
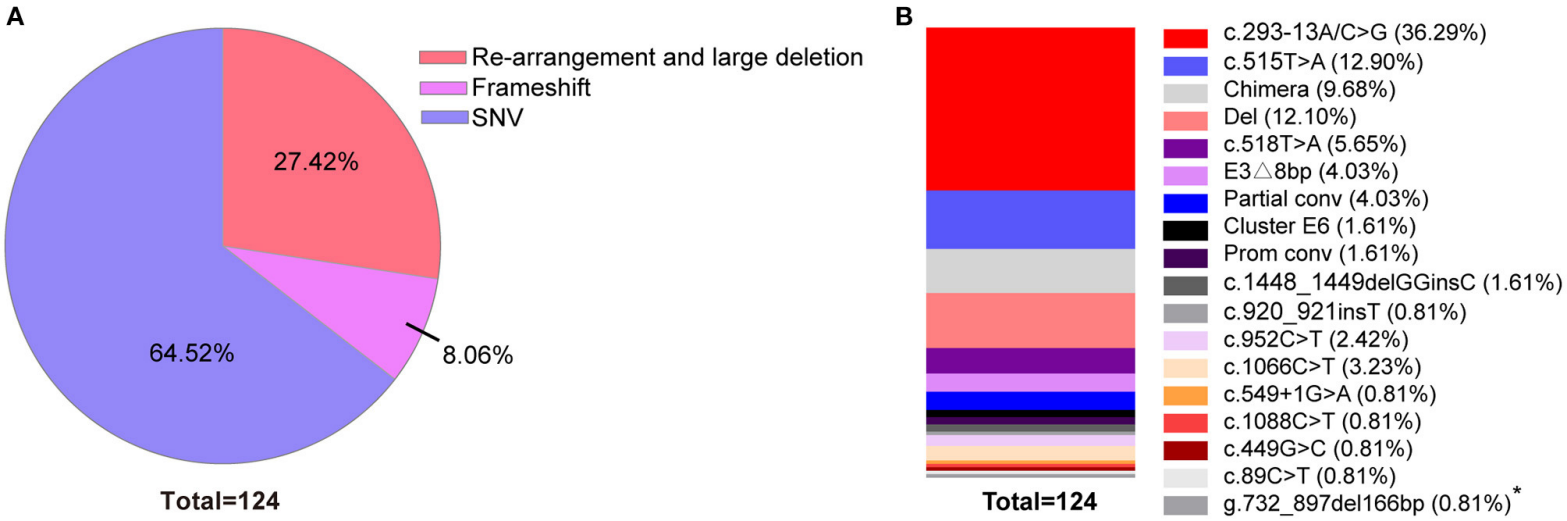
Percentage of variants across 59 classic 21-OHD patients. **(A)** The percentage of variants classified by re-arrangement and large deletion, frameshift and SNV. **(B)** The percentage of each variants shown by vertical slice-chart. (*n* = 124). *Variant site which not been reported to date.

Variant analysis was performed after clinical diagnosis at 1–3 months after birth. A total of 18 variants were found in the 59 patients. The most frequent variant was c.293-13A/C>G (36.29%). Rare pathogenic variants were also found that included c.549+1G>A and c.499G>C. 17 of the variants were reported before (Bidet et al., 2009; New et al., 2013; Hong et al., 2015; Wang et al., 2016; Concolino and Costella, 2018; Dundar et al., 2019). One novel mutation of small deletion was detected, g.732_897del166bp, which leads to partial deletion of exon 3 and intron 3 and suspected pathogenic mutation while the specific functional impact is unknown yet (Figure 3; Supplementary Figure 1).

### Relationship Between Genotype and Clinical Phenotype

**Figure 4:**
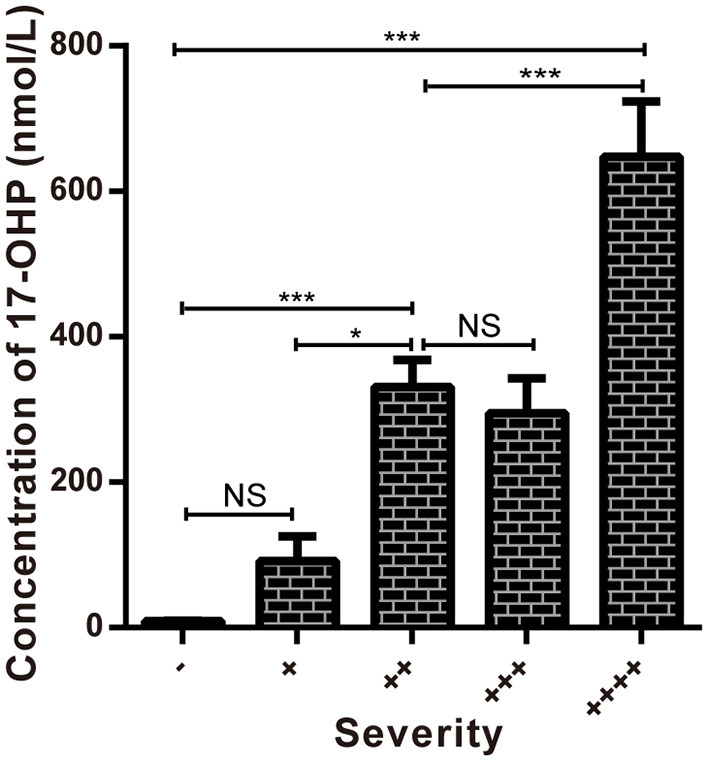
Comparison of the concentrations of 17-OHP in different severity groups. *P < 0.05 ***P < 0.001; NS, no significance.

Enzyme activity is highly correlated with clinical severity of 21-OHD and variants in the *CYP21A2* have differential impacts on enzyme activity (New et al., 2013). In our study, we combined the pattern, degree, and location of mutations with clinical test indicators and clinical manifestations to explore the relationship between different genotypes and clinical phenotypes of 21-OHD. We found that SW 21-OHD patients (28 males and 22 females) with different variants showed different degrees of clinical symptoms. Patients (six males) with large deletions or conversions of *CYP21A2* showed severe clinical manifestations including hyperpigmentation, vomiting, hypotension, hyponatremia, hyperkalemia, and shock, with a high level of 17-OHP (Table 4; Figure 4). Patients (11 males and 10 females) with partial deletion or conversion and point mutation, showed dehydration, hyperpigmentation, and some of the females had obvious ambiguous genitalia. Patients (three males and seven females) with frameshift and point mutations showed mild dehydration and hyperpigmentation. Females with these variants had different degrees of clitoromegaly. The coding impact of SV 21-OHD patients (six males and three females) was shown to be largely due to point mutations and the most frequent mutation detected as c.515T>A (p. I172N). Patients with SV 21-OHD showed mild hyperpigmentation, without vomiting, diarrhea or obvious genitals malformation with no significant increase in 17-OHP concentration.

**Table 4:** Variant distributions in 21-OHD patients.

**Index**	**Coding** **impact**	**Genotype**	**Variant** **location**	**17-OHP** **(nmol/L)**	**ACTH** **(pg/ml)**	**Cortisol** **(ug/dl)**	**T** **(ng/ml)**	**Na^**+**^** **(mmol/L)**	**K^**+**^** **(mmol/L)**	**Cl^**−**^** **(mmol/L)**	**CAH** **phenotype**	**Severity**	**Sex** **(Male/** **Female)**	**Patients proportion**
1	Large gene deletions and conversions	Del/Del		647.68 ± 169.62	124.49 ± 0.36	35.99 ± 62.07	27.79 ± 13.04	127.50 ± 124.85	6.45 ± 5.81	97.93 ± 97.21	SW	++++	2/0	2/59
2		Del/Chimera									SW	++++	2/0	2/59
3		Chimera/Cluster E6	Exon 6								SW	++++	1/0	1/59
4		Chimera/c.549+1G>A	Exon 4								SW	++++	1/0	1/59
5	Gene deletions or conversions and point mutations	Del/c.293-13A/C>G	Intron 2	303.97 ± 223.33	339.38 ± 239.19	16.47 ± 11.91	11.34 ± 16.75	124.60 ± 7.76	5.86 ± 1.08	97.28 ± 6.76	SW	+++	3/3	6/59
6		Del/c.952C>T	Exon 8								SW	+++	0/1	1/59
7		Del/c.1088C>T	Exon 8								SW	+++	0/1	1/59
8		Del/c.515T>A	Exon 4								SW	+++	1/0	1/59
9		Chimera/c.518T>A	Exon 4								SW	+++	3/0	3/59
10		Chimera/c.293-13A/C>G	Intron 2								SW	+++	1/2	3/59
11		Chimera/c. [293-13A/C>G; 952C>T]	Intron 2; Exon8								SW	+++	0/1	1/59
12		Chimera/c.515T>A	Exon 4								SW	+++	0/1	1/59
13		Partial conv/c.293-13A/C>G	Intron 2								SW	+++	3/0	3/59
14		Partial conv/ Prom conv; c. 293-13A/C>G	Promoter; Intron 2								SW	+++	0/1	1/59
15	Frameshift mutation and point mutation	g.732_897del166bp/ c.515T>A	Exon 3- Intron 3; Exon 4	288.70 ± 199.92	185.76 ± 135.10	8.63 ± 4.45	3.3 ± 2.52	132.84 ± 3.25	5.06 ± 0.65	104.5 ± 6.18	SW	++	1/0	1/59
16		c.1448_1449delGGinsC/c.293-13A/C>G	Exon10; Intron 2								SW	++	0/1	1/59
17		c.1448_1449delGGinsC/ c.515T>A	Exon10; Exon 4								SW	++	1/0	1/59
18		c.293-13A/C>G/c.293-13A/C>G; E3Δ8nt	Intron 2; Exon3								SW	++	1/0	1/59
19		E3Δ8nt/c.293-13A/C>G	Exon3; Intron 2								SW	++	0/3	3/59
20		c.920_921insT/c.515T>A	Exon7/4								SW	++	0/1	1/59
21		Prom conv; c.89C>T / c.293-13A/C>G; E3Δ8nt	Promoter; Intron 2; Exon 1/3								SW	++	0/1	1/59
22		Cluster E6/c.518T>A	Exon 4/6								SW	++	1/0	1/59
23	Point mutations	c.293-13A/C>G/c.293-13A/C>G	Intron 2	327.37 ± 154.88	202.57 ± 163.15	11.75 ± 8.60	6.29 ± 5.59	125.71 ± 12.21	5.70 ± 1.13	97.47 ± 8.09	SW	++	6/2	8/59
24														
25		c.293-13A/C>G/c.1066C>T	Intron 2; Exon 8								SW	++	0/2	2/59
26		c.293-13A/C>G/ c.952C>T	Intron 2; Exon 8								SW	++	1/0	1/59
27		c.518T>A/c.518T>A	Exon 4								SW	++	1/1	2/59
28		c.293-13A/C>G/c.515T>A	Intron 2; Exon 4	132.22 ± 105.79	61.38 ± 86.63	12.16 ± 1.03	2.94 ± 1.48	-	-	-	SV	+	4/2	6/59
29		c.515T>A/c.1066C>T	Exon 4/8								SV	+	2/0	2/59
30		c.515T>A/c.515T>A	Exon 4								SV	+	0/1	1/59

*Data are presented as the median ± standard deviation. Del, entire CYP21A2 gene deletion; Chimera, CYP21A1P/CYP21A2 chimeric gene, exon 1- exon 3; Partial conv, partial conversion; Prom conv, promoter conversion (g. [−126C>T;−113G>A;−110T>C;−103A>G]); E3Δ8nt, c.329_336delGAGACTAC; Cluster E6, c. [707T>A; 710T>A; 716T>A]. Severity of clinical manifestations is scaled (+ to ++++). These were defined as follows; Obvious hyperpigmentation of the skin over the entire body, vomiting, diarrhea, hypotension, hyponatremia, hyperkalemia, and shock (++++); Dehydration, hyperpigmentation and some of the females showing obvious ambiguous genitalia (+++); Mild dehydration and hyperpigmentation, some of the females showing different degrees clitoromegaly (++); Mild hyperpigmentation only around the perineum and areola, without vomiting, diarrhea or obvious genitals malformation (+)*.

According to the clinical severity of 21-OHD, the corresponding variants were sorted from the most to least severe and ranked accordingly from large gene deletions and conversions, frameshift variants, point mutations (c.293-13A/C>G; c.1066C>T; c.952C>T; c.518T>A) and c.515T>A (only found in SV patients).

### Clinical Follow-Up

In our study, glucocorticoid and mineralocorticoid replacement therapy were used for continuous treatment of 21-OHD patients who were followed up to allow the medication to be adjusted. In these patients, females with more severe 21-OHD also received genital reconstruction and had a good prognosis. The follow-up results showed that the condition of patients had significantly improved.

## Discussion

Neonatal screening for CAH has been successfully implemented in China and demonstrated to effectively prevent adrenal crisis, reduce mortality and reduce the harm associated with hyperandrogenism. Our research found that from 2000 to 2019, the number of newborns increased year by year along with an increased proportion of neonatal screening over the same period (Figure 1; Table 1). These observed trends further highlight the importance of neonatal screening and its increased use in providing the timely detection and treatment of newborn diseases. Also, improvements in newborn screening procedures will provide further information in the improved diagnosis and treatment of more newborn diseases.

Our data show that the incidence of CAH had obvious differences according to race (Khalid et al., 2012; New et al., 2013; Heather et al., 2015; Tsuji et al., 2015; Kopacek et al., 2017; Güran et al., 2019; Hou et al., 2019). For instance, the incidence of CAH in Tokyo, Japan has been reported as one in 19,859 (Tsuji et al., 2015) which is similar to the incidence in Nanjing, Jiangsu province of China (Table 1). This compares to levels in Great Britain of one in 166,667 and Turkey of one in 7787 (Khalid et al., 2012; Güran et al., 2019).

The 21-OHD enzyme is encoded by the *CYP21A2* gene (OMIM: 613815) located on chromosome 6p21.33 (Concolino et al., 2017; Simonetti et al., 2018). The cDNA is 2 kb long and the encoded protein is predicted to contain 494 amino acids. Approximately 30 kb from the *CYP21A2* there is a non-functional pseudogene, *CYP21A1P*. Both the functional gene and the pseudogene share a high level of nucleotide sequence homology of 98% in the exon regions and 96% homology in the introns (Gidlöf et al., 2014). To date, more than 1,300 genetic variants of *CYP21A2* have been reported (http://www.hgmd.cf.ac.uk) but only 230 have been shown to affect human health.

By analyzing the 17-OHP concentrations corresponding to 21-OHD patients of different severity, we found that the concentration of 17-OHP was significantly different between SW and SV patients (Tables 2, 4; Figure 4). Furthermore, in SW patients, we found that patients with large deletions and conversions of *CYP21A2* were correlated with severe clinical manifestations. These patients also had higher levels of 17-OHP compared to SW patients with other variants (Table 4; Figure 4). These data suggest the detection of 17-OHP can be used to infer the severity of clinical manifestations in SW patients.

Genotypes are closely related to clinical phenotypes (Khalid et al., 2012; Neocleous et al., 2018; Xu et al., 2019). In a report from Southern China, the most frequent mutation was c.293-13A/C>G (41.1%) (Hou et al., 2019) which is in agreement with our data on neonatal CAH screening (Figure 3B; Supplementary Figures 1B,D). However, European data show that the most frequent mutation was c.841G>T (23.9%), followed by c.293-13A/C>G (New et al., 2013) indicating that mutations of *CYP21A2* may also have different prevalence across different races.

In our study, we found that in SW cases, the most frequent variants were c.293-13A/C>G and deletions. In SV cases the most frequent variants were c.515T>A. Given the complexity of genetic mutations and the existence of pseudogenes, mutations cannot be accurately detected using a single technology and so we established a combined approach using three different technologies. Gene sequencing was used to detect small mutations, whilst locus-specific PCR and MLPA analysis were used to detect large deletions or gene conversion mutations.

The safety of long-term use of prednisone (PD) vs. hydrocortisone (HC) in the treatment of children with 21-OHD of CAH remains controversial (Riepe and Sippell, 2007; Ahmed et al., 2019). Several issues related to patient growth and the final height of children remain to be fully resolved (Bonfig, 2017). The increased risk of developing obesity is another possible consequence of hypercortisolism in children with CAH (Völkl et al., 2006). Our treatment was mainly based on the administration of intravenous hydrocortisone during adrenal crisis (mainly in the neonatal period with high potassium and low sodium) at a dose of 100 mg/kg twice a day and oral administration of 9α-flurocortisone. Hydrocortisone acetate (10–15 mg/m2, every 8 h) and 9α-flurocortisone were given orally after the condition had stabilized. Based on our clinical experience, the dose of hydrocortisone acetate should be kept at a low level and the normal high limit of 17-OHP should be maintained. Also, careful monitoring of blood hormone levels, growth rate and bone age should be performed.

A major issue in neonatal screening programs for 21-OHD with the detection of 17-OHP is the high rate of false-positives (Cavarzere et al., 2009; Fingerhut, 2009), particularly in preterm neonates (van der Kamp et al., 2005). Urinary steroid metabolite analysis using gas chromatography-mass spectrometry (GC-MS) is a suitable diagnostic tool to determine 17-hydroxyprogesterone levels (Speiser et al., 2018), however, this approach is not suitable for the rapid, high throughput analysis of large numbers of samples. Therefore, we explored the potential of adding more specific markers for joint detection based on 17-OHP, such as 21-Deoxycortisol (21-deoxy) (Jailer, 1953; Jailer et al., 1955; Miller, 2019). This method could dramatically reduce the rate of false-positives and could be a robust approach for large scale analysis in the future.

Another important issue concerning neonatal screening programs for 21-OHD is that the detection of 17-OHP cannot detect NC-CAH newborns. Individuals with the NC form may be compound heterozygous with one severe and one mild pathogenic variant. They may also be homozygous with two mild pathogenic variants in which 20–60% residual enzymatic activity can be preserved (Gidlöf et al., 2013). Enzyme activity is closely correlated with clinical severity (New et al., 2013). Patients with NC-CAH exhibit a mild phenotype and are rarely accompanied by an increase in 17-OHP during the neonatal period. In these cases, the clinical symptoms are also not obvious in newborns but are observed in adolescents and adults due to androgen excess including premature pubarche, acne, hirsutism, polycystic ovary syndrome, and subfertility (Marino et al., 2011). Further determination of NC-CAH requires investigation using ACTH-stimulation tests (Livadas et al., 2015). Due to a large number of newborns, it is difficult to achieve stimulation tests during newborn screening and this approach is yet to be widely implemented. Currently, there is no NC-CAH newborn screening program.

In contrast to classical CAH patients, adrenal replacement is not required as a therapy for NC-CAH patients. The management of excess androgens uses antiandrogens and oral contraceptives to improve long-term outcomes. Glucocorticosteroid (GCS) therapy can be used to obtain special outcomes such as restoration of fertility (Auchus and Arlt, 2013). To effectively treat children with CAH including patients with NC-CAH, the conditions should be detected as early as possible. Secondary screening using technologies such as next-generation sequencing should be carried out as early as possible to ensure more comprehensive and effective screening in neonatal diseases.

## Data Availability Statement

The original contributions presented in the study are included in the article/Supplementary Material, further inquiries can be directed to the corresponding authors.

## Ethics Statement

The studies involving human participants were reviewed and approved by Ethics Committee of Nanjing Maternity and Child Health Care Hospital affiliated with Nanjing Medical University. Written informed consent to participate in this study was provided by the participants' legal guardian/next of kin.

## Author Contributions

YS and TJ designed the research. XW analyzed data and wrote the manuscript with contributions from all of the authors. YW, DM, and ZZ carried out the screening tests. YL and PY contributed to the follow-up. All authors approved the final manuscript.

## Conflict of Interest

The authors declare that the research was conducted in the absence of any commercial or financial relationships that could be construed as a potential conflict of interest.
