# Supplementary material for: Neonatal Screening and Genotype-Phenotype Correlation of 21-Hydroxylase Deficiency in the Chinese Population
Source: Front Genet. 2021 Jan 22;11:623125. doi: 10.3389/fgene.2020.623125 (PMC7862715; doi:10.3389/fgene.2020.623125)
Supplement: Supplementary file 4 [file Data_Sheet_4.PDF]

Supplementary Material

1 Supplementary Figures and Tables

1.1 Supplementary Figures

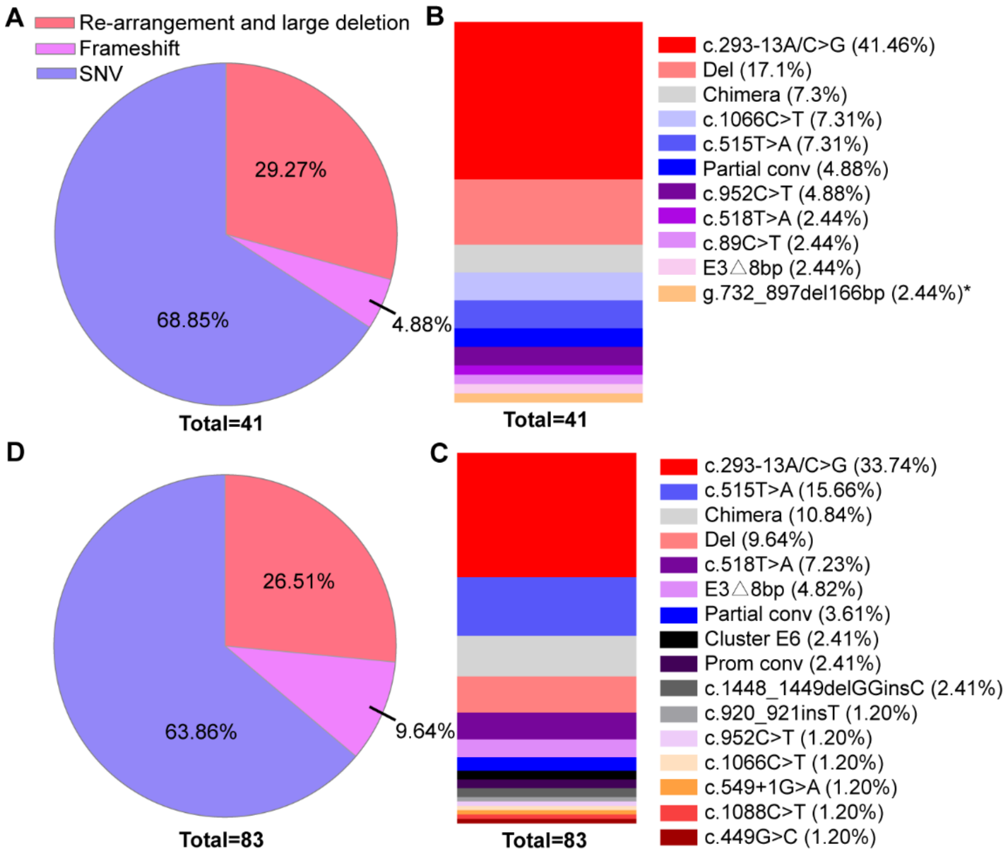

Supplementary Figure 1. The percentage of variants across classic 21-OHD patients. (**A-B**). 41 variants from 19 neonates born in Nanjing, Jiangsu province of China. (**C-D**). 83 mutations from 40 patients born in other cities in Eastern China. * Variant site which not been reported to date.

1.2 Supplementary Tables

Supplement Table 1. Primers for amplification of PCR products.

Designation	Sequence (5'-3')	Specificity	Location(nt)

**ME0008**	GCTTCTTGATGGGTGATCAAT	CYP21A2	−216 to −196[a]
**ME0066**	CCTCAATCCTCTGCAGCG	CYP21A2	3152 to 3169[a]
**ME0059**	TCCCCAATCCTTACTTTTTGT C	CYP21A1P	−840 to −819[b]
**ME0067**	CCTCAATCCTCTGCGGCA	CYP21A1P	3151 to 3168[b]
**E6wt**	AAGAGGGATCACATCGTGGAGAT	CYP21A2	1367 to 1389[a]
**E6mut**	AAGAGGGACCACAACGAGGAGAA	CYP21A1P	1370 to 1392[b]
**Tena32F**	CTGTGCCTGGCTATAGCAAGC	TNXB	78,918 to 78938[c]
**Tena36F2**	AGGCGCTCGCTATGAGGTGAC	TNXB	78,918 to 78938[c]
**XA36F**	AAGAGGGACCACAACGAGGAGAA	TNXA	4256 to 4276[d]

a, b, c and d based on GenBank accession number M12792.1, AL645922, AL049547 and S38953

Supplementary Table 2. Primer sets for amplification of the CYP21A2 and CYP21A1P and their possible chimeric forms.

**Primer set**	**Presumable PCR product**
**ME0008 and ME0066**	CYP21A2 (genuine gene)
**ME0059 and ME0067**	CYP21A1P (pseudogene)
**ME0059 and ME0066**	CYP21A1P/CYP21A2 (chimeric gene)
**ME0008 and ME0067**	CYP21A2/CYP21A1P (rearrangement product)
